# Risk factors for anastomotic leakage after laparoscopic low anterior resection with DST anastomosis

**DOI:** 10.1007/s00464-014-3564-0

**Published:** 2014-05-23

**Authors:** Kenji Kawada, Suguru Hasegawa, Koya Hida, Kenjiro Hirai, Kae Okoshi, Akinari Nomura, Junichiro Kawamura, Satoshi Nagayama, Yoshiharu Sakai

**Affiliations:** 1 Department of Surgery, Graduate School of Medicine, Kyoto University, 54 Shogoin-Kawara-cho, Sakyo-ku, Kyoto, 606-8507 Japan; 2 Department of Surgery, Japan Baptist Hospital, Kyoto, Japan; 3 Department of Surgery, Graduate School of Medicine, Saga University, Saga, Japan; 4 Department of Surgery, Shiga Medical Center for Adults, Moriyama, Japan; 5 Department of Gastroenterological Surgery, Gastroenterological Center, Cancer Institute Hospital, Japanese Foundation for Cancer Research, Tokyo, Japan

**Keywords:** Rectal cancer, Anastomotic leakage, Double-stapling technique, Laparoscopic low anterior resection

## Abstract

**Background:**

Laparoscopic rectal surgery involving rectal transection and anastomosis with stapling devices is technically difficult. The aim of this study was to evaluate the risk factors for anastomotic leakage (AL) after laparoscopic low anterior resection (LAR) with double-stapling technique (DST) anastomosis.

**Methods:**

This was a retrospective single-institution study of 154 rectal cancer patients who underwent laparoscopic LAR with DST anastomosis between June 2005 and August 2013. Patient-, tumor-, and surgery-related variables were examined by univariate and multivariate analyses. The outcome of interest was clinical AL.

**Results:**

The overall AL rate was 12.3 % (19/154). In univariate analysis, tumor size (*P* = 0.001), operative time (*P* = 0.049), intraoperative bleeding (*P* = 0.037), lateral lymph node dissection (*P* = 0.009), multiple firings of the linear stapler (*P* = 0.041), and precompression before stapler firings (*P* = 0.008) were significantly associated with AL. Multivariate analysis identified tumor size (odds ratio [OR] 4.01; 95 % confidence interval [CI] 1.25–12.89; *P* = 0.02) and precompression before stapler firings (OR 4.58; CI 1.22–17.20; *P* = 0.024) as independent risk factors for AL. In particular, precompression before stapler firing tended to reduce the AL occurring in early postoperative period.

**Conclusions:**

Using appropriate techniques, laparoscopic LAR with DST anastomosis can be performed safely without increasing the risk of AL. Important risk factors for AL were tumor size and precompression before stapler firings.

Total mesorectal excision (TME) was introduced by Heald in 1982 [1] and has been accepted as the standard technique for rectal surgery because it decreases local recurrence and improves functional results. Laparoscopic surgery for colon cancer was introduced in the 1990s, and has shown promising results. Laparoscopic low anterior resection (LAR) for rectal cancer is technically more difficult than laparoscopic colectomy for colon cancer because of the difficulties related to rectal transection and anastomosis within a narrow pelvic space. A higher incidence of positive circumferential margins after laparoscopic LAR was shown in an initial controlled trial [2], but an increasing number of recent studies have shown that laparoscopic surgery for rectal cancer is safe and feasible [3–7].

Anastomotic leakage (AL) is the most common complication after rectal cancer surgery and can result in not only increased morbidity and mortality but also increased local recurrence and poorer prognosis [8–10]. The double-stapling technique (DST) has greatly facilitated intestinal reconstruction especially for anastomosis after LAR. Despite technical improvements and instrumental developments, recent studies have reported that the AL rate remains at 6.3–13.7 %; the most commonly reported rate is approximately 10 % [7, 9, 11–14]. Risk factor analyses for AL after open LAR have been widely reported. However, a few studies have analyzed the risk factors for AL after laparoscopic LAR [14–18]. In addition, the rates of protective diverting stoma, preoperative chemoradiotherapy, and TME in each study were not consistent, which might produce different results. In the present study, cases with protective diverting stoma or preoperative chemoradiotherapy were excluded from the analysis to investigate the pure risk factor for AL.

We previously reported that precompression before stapler firings is a critical factor for gaining successful staple formation in an animal model [19]. To our knowledge, this is the first study to investigate the effect of precompression before stapler firings in a clinical setting. The aim of the present study was to identify the risk factors associated with AL in a single institution where standardized laparoscopic LAR with DST anastomosis was performed.

## Materials and methods

### Study population

A total of consecutive 162 patients underwent elective laparoscopic LAR with DST anastomosis at Kyoto University Hospital between June 2005 and August 2013. Among those patients, eight patients were excluded because they had the following factors: a tumor histopathology other than adenocarcinoma (*n* = 1); construction of protective diverting stoma (*n* = 4); conversion to open surgery (*n* = 3). Finally, a total of 154 patients with primary rectal cancers were included in this retrospective study. No patients had preoperative radiotherapy or chemoradiotherapy. The lower edge of the tumor was within 10 cm from the anal verge in all cases. Tumors located between the inferior margin of the second sacral vertebra and the peritoneal reflection were recorded as the upper rectum, while those located below the peritoneal reflection were recorded as the lower rectum [20]. The location of the tumor was determined by pelvic computed tomography, colonoscopy, and/or barium enema preoperatively and confirmed during surgery. The following patient-, tumor-, and surgery-related 25 variables were included in the analysis: patient-related [age, sex, body mass index (BMI), preoperative serum albumin and hemoglobin levels, preoperative chemotherapy], tumor-related (tumor location, maximum tumor diameter, UICC-TNM stage (7th edition) [21], lymphatic invasion, venous invasion), and surgery-related (operative time, intraoperative bleeding, level of inferior mesenteric artery (IMA) ligation, lateral lymph node dissection, simultaneous resection of other organs, number of cartridges of the linear stapler used for rectal transection, size of the circular stapler, height of the anastomosis from the anal verge, removal of crossing point where two staple lines intersected, precompression before stapler firings, placement of a pelvic drain, placement of a transanal tube). Written informed consent was obtained from all patients for the use of their clinical data in the future.

### Surgical method

All procedures were conducted by well-experienced, board-certified laparoscopic colorectal surgeons at our institution. All patients received standard bowel preparation and antibiotic prophylaxis. The surgical technique was standardized, as described previously [22, 23]. High ligation of IMA was routinely performed, although low ligation of IMA (preservation of left colic artery) was performed depending on the condition of the patient’s blood vessel. The splenic flexure was mobilized totally or partially, depending on the bowel length. After mobilization of the left colon, tumor-specific mesorectal excision, including TME (according to the tumor location), was performed as the standard surgical technique. The main principle of this technique is sharp mesorectal dissection with a nerve-preserving technique. After clamping distal to the tumor to allow washout of the rectal stump, the rectum was transected using the linear stapler (Echelon 60 or Endo-Cutter, Ethicon Endo-Surgery, Cincinnati, OH, USA). After the surgical specimens were removed through the small incision, the anvil of the circular stapler was positioned in the proximal colon. The circular stapler (CDH, Ethicon) was inserted though the rectum, and then end-to-end DST anastomosis was completed intracorporeally. The “doughnut” created after anastomosis was inspected for completeness. Air-tightness was routinely tested by the transanal instillation of air. The height of anastomosis from the anal verge was measured by the digital rectal examination during anesthesia. Cases with protective diverting stoma were excluded. Cases converted to a transanal hand-sewn coloanal anastomosis were also excluded.

### Definition of clinical anastomotic leakage

Clinical leakage signs were defined as abdominal pain, fever, pus, or fecal discharge from the pelvic drain, peritonitis, and pelvic abscess. All clinically suspicious symptoms were confirmed by digital rectal examination, sigmoidoscopy and radiographic examination (e.g., extravasation of endoluminally administered water-soluble contrast enema, abscess at the level of anastomosis, and fluid/air bubbles surrounding the anastomosis on computed tomography). The diagnosis of AL was done within 30 days after surgery. Using the proposed grading system [24], AL was classified into three grades: grade A required no active therapeutic intervention; grade B required active therapeutic intervention; and grade C required re-operation. We included symptomatic AL (grade B and C) for primary endpoint analysis. Asymptomatic AL (grade A) was not considered, because routine contrast enemas were not performed after surgery in our institution.

### Statistical analysis

All statistical analyses were performed using the SPSS software, version 11.50 (SPSS Inc, Chicago, IL, USA). The Chi-square test, Fisher’s exact test, and Mann–Whitney *U* test were used for categorical variables comparison and analysis. All analyses were two-sided, and a *P* value of <0.05 was considered statistically significant. To determine factors associated with AL, multivariate logistic regression analysis was used and factors with a *P* value of <0.05 were included in the model.

## Results

### Patients population

In total, consecutive 162 patients underwent elective laparoscopic LAR with end-to-end DST anastomosis. To investigate the pure risk factors of AL, patients with the following factors were excluded: a tumor histopathology other than adenocarcinoma (*n* = 1), construction of protective diverting ileostomy (*n* = 4), and conversion to open surgery (*n* = 3). Therefore, a total of 154 patients were enrolled for analysis. Patient characteristics are listed in Table 1. Among 154 patients, 111 (72.1 %) were male and 43 (27.9 %) were female. The median age was 66 years old (range 36–88). Their median BMI was 21.6 (range 10.5–30.0). The lower edge of the tumor was within 10 cm from the anal verge in all cases. A total of 101 patients (65.6 %) had the upper rectal cancer, and the remaining 53 patients (34.4 %) had the lower rectal cancer. Preoperative chemotherapy was performed in 25 patients (16.2 %). Preoperative chemoradiotherapy was not performed in this series because of construction of a protective diverting stoma.

**Table 1:** Patient and tumor characteristics (*n* = 154)

Characteristics		No. of Patients
Age (years)		
Median ± SD (range)	66 ± 9.9 (36–88)	
BMI (kg/m^2^)		
Median ± SD (range)	21.6 ± 3.2 (10.5–30.0)	
Sex		
Male		111
Female		43
Location		
Upper		101
Lower		53
UICC-TNM Stage		
0		2
I		45
II		61
III		34
IV		12
T category		
Tis		2
T1		17
T2		34
T3		81
T4		20
N category		
N0		110
N1		26
N2		18
M category		
M0		142
M1		12
Tumor size (mm)		
Median ± SD	40 ± 19	
Preoperative chemotherapy		25
Anastomotic leakage		19

### Anastomotic leakage

Among 154 patients, symptomatic AL occurred in 19 patients (12.3 %): 15 were male and 4 were female. Their median BMI was 22.1 (range 17.0–27.3). The AL rate was 11.9 % (12/101) in patients with upper rectal cancer and 13.2 % (7/53) in patients with lower rectal cancer. AL requiring re-operation (grade C) occurred in 8 cases (5.2 %: 8/154); diverting stoma in 6 cases, Hartmann procedure in one case, and drainage in one case. AL not requiring re-operation (grade B) occurred in 11 cases (7.1 %: 11/154); treated by transanal drainage [25] in seven cases, and by antibiotics in four cases. The median time at which AL was confirmed was postoperative day (POD) 6 (range 2–15). Fistula formation with vesicle and vagina occurred in three cases and one case, respectively. The median time to hospital discharge was POD 45 (range 16–85), and there was no death related to AL (Table 2).

**Table 2:** Clinical features of 19 patients with AL

Characteristics		No. of Patients
Age (years)		
Median (range)	65 (41–80)	
BMI (kg/m^2^)		
Median (range)	22.1 (17.0–27.3)	
Sex		
Male		15
Female		4
Location		
Upper		12
Lower		7
Detection time (day)		
Median (range)	POD 6 (2–15)	
Grade		
B		12
C		7
Treatment		
Diverting ileostomy		6
Hartmann procedure		1
Drainage		1
Transanal drainage		7
Antibiotics		4
Fistula		
Rectovesical fistula		3
Rectovaginal fistula		1
Length of hospital stay		
Median (range)	POD 45 (16–85)	
Mortality		0

### Risk factors related to DST anastomotic leakage

On univariate analysis, symptomatic AL was significantly associated with tumor size (≥5.0 cm), operative time (≥5.0 h), operative bleeding (≥100 ml), lateral lymph node dissection, multiple firings of the linear stapler (≥3 firings), and precompression before stapler firings (Tables 3, 4). In addition, there was a tendency for placement of a transanal tube to reduce AL, with *P* value less than 0.10. No significant differences were found in terms of age, sex, BMI, preoperative serum albumin and hemoglobin levels, preoperative chemotherapy, tumor location, UICC-TNM stage, lymphatic invasion, venous invasion, level of IMA ligation, simultaneous resection of other organs, height of the anastomosis, removal of crossing point where two staple lines intersected, size of the circular stapler, and placement of a pelvic drain. In the precompression group, we secured more than 30-s intervals before each firing of the linear stapler, and more than 2-min interval before firing of the circular stapler, while we did not secure such enough precompression time in the non-precompression group. We previously reported that precompression before stapler firings is a critical factor for successful staple formation in an animal model [19]. Therefore, we analyzed the effect of precompression before stapler firings in this clinical setting, and found that it significantly reduced the AL rate (28.6 % in the non-precompression group vs. 8.7 % in the precompression group; *P* = 0.008).

**Table 3:** Univariate analysis of patient/tumor-related factors

Variables	Patients with AL		
	*n*	%	*P* value
Age (years)			0.43
<70	15/107	14.0	
≥70	4/47	8.5	
Sex			0.59
Male	15/111	13.5	
Female	4/43	9.3	
BMI (kg/m^2^)			0.75
<25	15/127	11.8	
≥25	4/27	14.8	
Albumin (g/dl)			0.59
<3.5	0/8	0.0	
≥3.5	19/146	13.0	
Hemoglobin (g/dl)			1
<11	2/15	13.3	
≥11	17/139	12.2	
Location			0.80
Upper	12/101	11.9	
Lower	7/53	13.2	
Tumor size (cm)			0.001
<5.0	7/111	6.3	
≥5.0	12/43	27.9	
T category			1
Tis, T1, T2	6/53	11.3	
T3, T4	13/101	12.8	
N category			1
N0	14/110	12.7	
N1, N2	5/44	11.4	
UICC-TNM Stage			1
I, II	13/103	12.6	
III, IV	6/51	11.8	
Lymphatic invasion			1
Negative	13/103	12.6	
Positive	6/51	11.8	
Venous invasion			1
Negative	8/67	11.9	
Positive	11/87	12.6	
Preoperative chemotherapy			0.52
No	15/129	11.6	
Yes	4/25	16.0

**Table 4:** Univariate analysis of surgery-related factors

Variables	Patients with AL		
	*n*	%	*P* value
Operative time (min)			0.049
<300	7/90	7.8	
≥300	12/64	18.7	
Intraoperative bleeding (ml)			0.037
<100	11/120	9.2	
≥100	8/34	23.5	
Ligation of IMA			0.29
High ligation	15/133	11.3	
Low ligation	4/21	19.0	
Lateral lymph node dissection			0.009
No	15/146	10.3	
Yes	4/8	50.0	
Simultaneous resection			0.60
No	19/147	12.9	
Yes	0/7	0.0	
Anastomosis level from anal verge (mm)			0.27
<30	4/25	16.0	
≥30	9/107	8.4	
Number of cartridges for rectal transection			0.041
1.2	13/131	9.9	
≥3	6/23	26.0	
Crossing point of staple lines			0.29
Absent	11/106	10.4	
Present	8/46	17.4	
Precompression before stapler firings			0.008
No	8/28	28.6	
Yes	11/126	8.7	
Diameter of circular stapler (mm)			1
25	1/16	6.3	
29	13/121	10.7	
Placement of a pelvic drain			0.18
No	5/24	20.8	
Yes	14/130	10.8	
Placement of a transanal tube			0.096
No	6/26	23.1	
Yes	13/128	10.2	

In the multivariate analysis including factors with a *P* value of ≤ 0.05, only tumor size (≥5.0 cm) and precompression before stapler firings remained significantly correlated with AL (Table 5; odds ratio [OR] 4.01; 95 % confidence interval [CI] 1.25–12.89; *P* = 0.02 and OR 4.58; CI 1.22–17.20; *P* = 0.024, respectively).

**Table 5:** Multivariate analysis of risk factors associated with AR

Variables	OR	95 % CI	*P* value
Tumor size (≥5 cm)	4.01	1.25–12.89	0.020
Operative time (≥300 min)	2.9	0.77–11.14	0.114
Intraoperative bleeding (≥100 ml)	0.88	0.23–3.31	0.849
Lateral lymph node dissection (yes)	3.67	0.63–21.34	0.148
Number of cartridges for rectal transection (≥3)	0.90	0.22–3.71	0.887
Precompression before stapler firings (no)	4.58	1.22–17.20	0.024

*OR* odds ratio, *CI* confidence interval

Based on the timing to be confirmed AL, 19 patients with developing AL were classified into two groups; the early leakage group (POD 5 or less; *n* = 8) and the late leakage group (POD more than 5; *n* = 11) (Table 6). Regarding the severity of AL, grade C occurred in 50 % (4/8) of the early leakage group, whereas in 36.3 % (4/11) of the late leakage group. Emergency operation was needed due to major leakage in 37.5 % (3/8) of the early leakage group, whereas in 18.2 % (2/11) of the late leakage group. Importantly, precompression before stapler firings tended to reduce the early leakage compared with the late leakage (25 % (2/8) and 81.8 % (9/11), respectively). In addition, multiple firings of the linear stapler (≥3 firings) also tended to be associated with the early leakage compared with the late leakage (62.5 % (5/8) and 9.1 % (1/11), respectively).

**Table 6:** Type of AL

Variables	Early leakage (*n* = 8)	Late leakage (*n* = 11)
Detection time		
Median ± SD, POD days	3.5 ± 1.4	10 ± 3.6
Grade		
B	4	7
C	4	4
Emergency operation		
No	5	9
Yes	3	2
Tumor size		
Median ± SD (mm)	63 ± 17	48 ± 17
Anastomosis level from anal verge		
Median ± SD (mm)	28 ± 16	42 ± 23
Operative time (min)		
<300	4	3
≥300	4	8
Intraoperative bleeding (ml)		
<100	4	7
≥100	4	4
Lateral lymph node dissection		
No	6	9
Yes	2	2
Number of cartridges for rectal transection		
1.2	3	10
≥3	5	1
Precompression before stapler firings		
No	6	9
Yes	2	2
Placement of a transanal tube		
No	4	2
Yes	4	9

## Discussion

AL is a major problem in patients who undergo operations for rectal cancers. It is associated with not only postoperative morbidity and mortality, but also local recurrence and patient’s survival [8–10]. Several risk factors, including age, sex, intraoperative bleeding, obesity, preoperative chemoradiotherapy, protective diverting stoma, pelvic drainage, tumor size, tumor location, and the level of anastomosis, have been reported to be associated with AL after open LAR [11, 26–29]. In contrast, only a few studies have examined risk factors for AL after laparoscopic LAR [14–18]. Several studies reported that laparoscopic surgery and open surgery for rectal cancer did not differ in terms of the AL rate [2, 3, 5, 30]. Laparoscopic rectal surgery provides an excellent operative field in a narrow pelvic space, and enables the preservation of autonomic nervous system more precisely. However, rectal transection using a laparoscopic linear stapler is relatively difficult when compared with open surgery because of the width and limited performance of the linear stapler. The devices and techniques used for laparoscopic LAR are different from those used for open LAR, which suggests that the risk factors for AL after laparoscopic LAR may also differ from those after open LAR. In the present study, multivariate analysis identified tumor size (≥5.0 cm) and precompression before stapler firings as independent risk factors of symptomatic AL after laparoscopic LAR with DST anastomosis (Table 5; *P* = 0.02 and 0.024, respectively). Tumor size is well known to be a risk factor for AL after LAR [29]. Pelvic space is limited, and so tumor size could adversely affect the ease of rectal transection and anastomosis. We previously reported that a sufficient amount of precompression time before stapler firings resulted in reduced intestinal wall thickness and proper staple formation in an animal model [19], which was in agreement with the result of this clinical study. This study provided the first evidence that precompression before stapler firings was associated with AL in a clinical setting. We assume that precompression time and proper cartridge selection according to the wall thickness were critical to achieve secure staple formation.

Previous studies reported that the use of more than three cartridges for rectal transection was a risk factor for AL after laparoscopic LAR [14, 15, 17]. When the number of stapler cartridges increases, there is a concern that an increased number of stapler firings may lead to small defects between the staple lines and, in turn, cause AL. In the present study, AL occurred in 26.0 % (6/23) of the cases in which more than three cartridges were used, whereas in only 9.9 % (13/131) of the cases in which one or two cartridges were used (Table 4; *P* = 0.041). In addition, the AL rate in cases with two cartridges was 10.9 % (11/101), whereas that in cases with one cartridge was 6.7 % (2/30). Although there was no statistical significance in multivariate analysis (Table 5), we assume that the efforts to reduce the number of linear stapler seem to be recommended.

Several surgical techniques for laparoscopic LAR have been proposed to decrease AL. Ito et al. [15] reported that vertical rectal transection through an additional suprapubic site was useful for avoiding multiple stapler firings and decreasing the AL rate. Kuroyanagi et al. [23] reported that rectal transection was performed using two cartridges in most cases, with harmonious operator-assistant movement. They insisted the technical efforts to remove the crossing point of staple lines, which might otherwise be the cause of AL. In the present study, we analyzed whether the remnant crossing point could increase the AL rate, and found that it was not significantly associated with AL (Table 4); AL occurred in 17.4 % (8/46) of cases with remnant crossing point, whereas in 10.4 % (11/106) of cases without remnant crossing point (*P* = 0.29). We assume that surgeons do not have to persist to remove the crossing point, especially when the crossing point is placed near the edge of the rectal stump and so removal of the crossing point is technically difficult. To our knowledge, this is the first study to investigate the effect of the remnant crossing point in a clinical setting.

Some studies recently reported that a transanal tube was important to prevent AL after LAR [31, 32], although other study reported that a transanal stent did not reduce AL [33]. In theory, a transanal tube decreases the intraluminal pressure around the anastomotic site, and protects the anastomosis from watery stool and flatus when gastrointestinal motility improves. In the present study, AL occurred in 10.2 % (13/128) of cases with a transanal tube, whereas in 23.1 % (6/26) of cases without a transanal tube (Table 4; *P* = 0.096). Although there was no statistical significance, we assume that a transanal tube seems to be useful to reduce the AL rate. We usually remove a transanal tube at 5–7 days after surgery.

A number of studies have reported that lower anastomosis level is an important risk factor for AL after LAR [27, 28]. However, the correlation between anastomosis level and AL was not statistically significant in the present study: AL rates for low anastomosis (height of the anastomosis from the anal verge was less than 3 cm) and high anastomosis (height of the anastomosis from the anal verge was 3 cm or more) were 16.0 % (4/25) and 8.4 % (9/107), respectively (Table 4; *P* = 0.27). In addition, the correlation between tumor location and AL was not significant (Table 3; *P* = 0.80). Although there was no statistical significance, the height of the anastomosis or the tumor location can reflect technical difficulties of laparoscopic LAR. All surgeries in the present study were conducted by well-experienced, board-certified laparoscopic colorectal surgeons. This minimized the risk of bias potentially associated with the early phase of the learning curve of surgeons, and with any inter-institutional variability in a multi-institutional trial.

There is still debate as to whether the creation of diverting stoma reduces AL. A recent randomized controlled study showed that the creation of diverting stoma reduced the incidence and clinical significance of AL [34]. A considerable amount of retrospective studies have also described the beneficial effect of a diverting stoma on AL [11, 35, 36]. On the other hand, there are some studies that the creation of a diverting stoma did not reduce the AL rate [37, 38]. However, it is generally agreed that the creation of a diverting stoma can reduce the incidence of the severe complications that AL can cause. In the present study, cases with a diverting stoma were excluded from the analysis, because the creation of a diverting stoma seems to effectively reduce the clinical significance of AL and could be considered in high-risk patients.

In conclusion, we demonstrated that tumor size and precompression before stapler firings were independent risk factors for AL after laparoscopic LAR with DST anastomosis. In addition, precompression before stapler firings and multiple firings of the linear stapler tended to be associated with the AL occurring in early postoperative period. This study provides interesting data in the effort to reduce AL. However, because of the retrospective nature, the limited number of patients, and the likely multifactorial nature of AL, it is hard to draw robust conclusions. The outcomes of this study could not be corrected in a case-mix adjusted comparison, since this requires a large amount of cases to prevent over-fitting. Further studies including a large multi-institutional randomized controlled study are required to identify risk factors of AL and to develop the approaches to reduce this risk for patients with rectal cancers who undergo laparoscopic LAR.
